# Ashwagandha attenuates TNF-α- and LPS-induced NF-κB activation and CCL2 and CCL5 gene expression in NRK-52E cells

**DOI:** 10.1186/s12906-015-0958-z

**Published:** 2015-12-15

**Authors:** Elizabeth Grunz-Borgmann, Valeri Mossine, Kevin Fritsche, Alan R. Parrish

**Affiliations:** Department of Medical Pharmacology and Physiology, School of Medicine, University of Missouri, MA 415 Medical Sciences Building, One Hospital Drive, 65212 Columbia, MO USA; Department of Biochemistry, University of Missouri, Columbia, USA; Division of Animal Sciences, University of Missouri, Columbia, USA

**Keywords:** Ashwagandha, CCL2, CCL5, Kidney, NF-κB

## Abstract

**Background:**

The aging kidney is marked by a chronic inflammation, which may exacerbate the progression of renal dysfunction, as well as increase the susceptibility to acute injury. The identification of strategies to alleviate inflammation may have translational impact to attenuate kidney disease.

**Methods:**

We tested the potential of ashwaganda, sutherlandia and elderberry on tumor necrosis factor-α (TNF-α) and lipopolysaccharide (LPS) induced chemokine (CCL2 and CCL5) expression in vitro.

**Results:**

Elderberry water-soluble extract (WSE) was pro-inflammatory, while sutherlandia WSE only partially attenuated the TNF-α-induced changes in CCL5. However, ashwaganda WSE completely prevented TNF-α-induced increases in CCL5, while attenuating the increase in CCL2 expression and NF-κB activation. The same pattern of ashwagandha protection was seen using LPS as the pro-inflammatory stimuli.

**Conclusions:**

Taken together, these results demonstrate the ashwaganda WSE as a valid candidate for evaluation of therapeutic potential for the treatment of chronic renal dysfunction.

## Background

Inflammation is a key component of the aging kidney [1]; in both human [2, 3] and animal studies from our laboratory [4], it has been shown that a significant number of genes whose expression is increased in aging are pro-inflammatory. The kidney may be uniquely sensitive to inflammation as it is a source for cytokine and chemokine synthesis within the tubular epithelium [5], and due to the high blood flow, it is continually exposed to circulating pro-inflammatory mediators. There are significant data linking inflammation to the loss of renal function [6, 7]; the pro-inflammatory environment may also be one of the mechanisms contributing to the increased severity of acute injury in the aging kidney [8].

Fibrosis is an accumulation of matrix, predominantly collagen, which is associated with loss of organ function as normal tissue is replaced by scar tissue [9]. The kidney is a prototypical example of an organ in which progressive fibrosis leads to organ failure [10, 11]. A signaling pathway that links inflammation and fibrosis in the kidney is NF-κB. This transcription factor has a long-recognized role as a pro-inflammatory mediator [12, 13]. A number of stimuli induce NF-κB activity in the kidney, including TNF [14] and angiotensin II [15] – both of which are associated with chronic kidney disease (CKD) [16, 17]. The list of NF-κB target genes is lengthy but includes CCL2 and CCL5 (RANTES) [18, 19].

CCL5 is a chemoattractant for monocytes [20]; CCR1 and CCR5 are receptors for CCL5 [21]. A number of studies now link CCL5, and other chemokines, including CCL21 and its receptor CCR7, to renal fibrosis. There is a parallel increase in the gene expression of CCL5 and collagen 1α1 in a mouse model of progressive fibrosis [22]. In a rat model of CKD, attenuation of inflammation is associated with decreased CCL5 expression and reduced fibrosis [23]. A CCR1 antagonist (BX471) is associated with reduction in fibrosis in multiple animal models [24, 25]. CCL2 (monocyte chemotactic protein-1; MCP-1) is a C-C chemokine that is a ligand for the CCR2 receptor and is a potent chemoattractant for leukocytes, T lymphocytes and NK (natural killer) cells [26]. Elevated urinary CCL2 is associated with the development of fibrosis in human renal allografts and may be a poor predictor of outcome [27, 28]. Thus, elevation of CCL2 and CCL5 may contribute the progressive fibrosis important in CKD.

Published data demonstrates that sutherlandia [29] and elderberry [30, 31] have anti-inflammatory effects. It is unclear as to whether these effects extend to renoprotection and, in fact, an elderberry-rich extract enriched in anthocyanins did not improve renal function in a small clinical trial of postmenopausal women [32]. In contrast, ashwagandha (*Withania somnifera*) has been demonstrated to be renoprotective via both antioxidant [33–35] and anti-inflammatory effects [36]. The current studies were designed to screen water-soluble extracts of ashwaganda, sutherlandia and elderberry for anti-inflammatory effects in renal epithelial cells.

## Methods

### Cell culture

NRK-52E cells (ATCC #CRL-1571) were grown in Dulbecco’s modified eagle medium F-12 (DMEM/F12, 1:1) without phenol red, but with L-glutamine and 15 mM HEPES (Gibco #11039-021). The growth media also contained 5 % fetal bovine serum (FBS; Atlanta Biologicals #S11150), 50 U/ml penicillin, and 50 mg/ml streptomycin (Gibco #15140-122). A NRK-52E:NF-κB reporter cell line was generated by stably transposing the insulated NF-κB pathway reporter DNA [37] into the chromatin of NRK-52E cells. The stable transfects were selected in DMEM/F12 containing 5 % FBS, and 1 μg/ml puromycin dihydrochloride (Sigma #P9620-10 ml). Both cell types were cultured at 37 °C in 5 % CO_2_. Cells were washed with 1X Dulbecco’s phosphate buffered saline (DPBS; Gibco #14190-144), harvested with TrypLE Express (Gibco #12604-021), and centrifuged at 1,500xrpm for 5 min at room temperature (RT).

### Dosing

An aqueous extract of *Sutherlandia frutescens* was prepared using the method described by Fernandes [38]. Briefly, 10 g of *S. frutescens* (ground powder of vegetative parts of *S. frutescens* purchased from Big Tree Nutraceutical, Fish Hoek, South Africa) was added to 250 mL of boiling water. This mixture was kept in a 100 °C water bath for 1 h, with stirring every 10 min. This mixture was allowed to cool overnight, in the dark. The decoction was transferred into 50 mL sterilized centrifuge tubes, and centrifuged at 2000×g for 20 min. The supernatant was sterile-filtered using a 0.2 μm nylon filter (Fisher Scientific, Pittsburgh, PA, USA), and then stored in 1.0 mL aliquots at −80 °C until used. The product identity was confirmed using HPLC/ELSD and HPLC/UV [39], which determined that the *S. frutescens* used in this study contained 3.3 % (w/w) of sutherlandioside B, a specific biomarker of this medicinal plant [39, 40].

The Ashwagandha used for these experiments was obtained from a commercial supplement (Now Foods, Bloomingdale, IL, USA). Each capsule contained 450 mg of a standardized extract containing a minimum of 2.5 % total withanolides. The contents of several capsules were weighed and dissolved in endotoxin-free, double-distilled water at a 1:10 ratio (wt/vol). After vigorous mixing this solution was allowed to sit overnight in the dark. The following morning the mixture was vigorously mixed again for 1 min, then non-soluble components were removed by centrifugation (2000×g for 20 min). The supernatant was sterile-filtered using a 0.2 μm nylon filter, and then stored in 1.0 mL aliquots at −80 °C until used.

The elderberry juice used in these experiments was obtained from elderberries harvested from North American varieties (*Sambucus nigra,* L. subsp. *canadensis (*L. Bolli) near the peak of ripeness and immediately frozen. Andrew L. Thomas (University of Misosuri) collected the specimens from Eridu Farm, Hartsburg (Boone County), MO, in full bloom on June 7, 2012. Location 38.710512 lat., −92.316186 long. A voucher specimen (A. Thomas 120) from the field was preserved at the University of Missouri (UMO) [41]. While frozen the berries were manually separated from stems, leaves, insects, and other debris. Berries were thawed, juiced, then re-frozen. At a later time, the frozen juice was thawed, centrifuged (1000×g for 30 min), then the clarified juice was filtered through a 0.2 μm sterile, pyrogen-free nylon filter (Fisher Scientific, Inc.) and stored at −80 °C in 100 μL aliquots. Just prior to addition to cell culture medium, the frozen, sterile-filtered juice was thawed and diluted 1000-fold so as to supply a final concentration of ~100 μg of juice solids/mL.

The presence and concentration of endotoxin in each of the extracts was quantified using the recombinant factor C endotoxin detection assay (Lonza Walkersville, Inc., Cat. 50-658U, Walkersville, MD, USA) following the instructions provided by the manufacturer. The principle of this kit is that the recombinant Factor C is activated by endotoxin binding, and the active moiety created then acts to cleave a synthetic substrate resulting in the generation of a fluorogenic compound. The highest concentration of endotoxin was found in the *S. frutescens* (i.e., ~0.8 ng LPS equivalents/mL), while the Ashwagandha extract and elderberry juice contained 0.004 and <0.001 ng of LPS equivalents/mL, respectively.

Recombinant Human Tumor Necrosis Factor alpha 1α (TNF-α; #rhtnf-α) and standard lipopolysaccharide from *E. coli* 0111:B4 strain- TLR4 ligand (LPS; #tlrl-eblps) were purchased from Invivogen. NRK-52E cells were plated at a density of 120,000 cells/well in 6-well plates and left to grow to confluency overnight. Cells were washed with 1X DPBS and then treated with water or botanical extract (1:100 dilution) for 24 h. Wells were washed and then treated with water, TNF-α or LPS, botanical extract, or a combination of TNF-α or LPS plus botanical extract for an additional 24 h. After 48 h treatment, cells were harvested for RNA extraction.

### NF−κB activity assay

NRK-52E:NF-κB cells were plated at a density of 100,000 cells/well in 12-well plates and left to grow to confluency overnight. They were dosed as described above. Media was removed and cells were washed twice with 1X DPBS. Reporter lysis buffer was added to each well and the plates were allowed to rock for 15 min at RT, followed by a freeze/thaw cycle for 30 min at −80 °C and 30 min rocking at RT. Cell lysates were collected and centrifuged for 15 s. Lysates were transferred to a new tube and assayed for NF-κB green fluorescent protein (GFP)/luciferase activity using the Luciferase Assay System kit (#E4030) from Promega. Briefly, 100 μl of lysate was placed into each well of a 96-well flat bottom, opaque white plate. The fluorescence of samples was read for GFP expression at 485/20 nm excitation and 528/20 nm emission wavelengths. Then the luciferase assay reagent was added to each well and the luminescence of the wells was read for luciferase expression on a Synergy HT plate reader using Gen5 (version 1.11) software from Bio-TEK. NF-κB activation is expressed as the individual luciferase/GFP values divided by the average control luciferase/GFP value.

### Real-time PCR

RNA was extracted from the cells using the NucleoSpin RNA kit (Macherey-Nagel #740955.50), with on-column DNase digestion. RNA was quantified by a spectrophotometric read using the Nanodrop 2000c (Thermo Scientific). cDNA was made from the quantified RNA using the High Capacity cDNA Reverse Transcription kit (Applied Biosystems #4368814), including a RNase inhibition step (Applied Biosystems #N8080119). Real-Time PCR was performed in duplicate using 200 ng cDNA/reaction in conjunction with Taqman assays (Applied Biosystems) using TaqProbe 2X qPCR Multiplex Master Mix (Bullseye #BEQPCR-PM) on the CFX96 Touch system (Bio-Rad). The following cycling conditions were followed: 95 °C for 20 s, then 95 °C for 1 s and 60 °C for 20 s repeated 40 times. TaqMan primer sets (Life Technologies) were used to determine Chemokine (C-C motif) ligand 2 (CCL2; RN00580555_m1) and chemokine (C-C motif) ligand 5 (CCL5; Rn00579590_m1). The Pfaffl method was used to determine relative quantitation, normalizing the data to cancer susceptibility candidate gene 3 (Casc3; RN00595941_m1) [42].

### Statistics

Data is expressed as the mean +/- standard error. A two-tailed *t*-test, assuming two-sample equal variance, was used to determine significance. *P*-values of <0.05 were considered statistically significant.

## Results

**Fig. 1:**
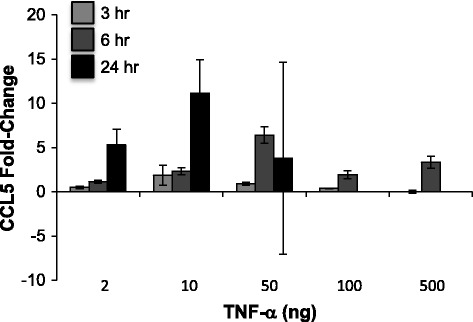
TNF-α induces CCL5 expression in NRK-52E cells. Confluent cells were challenged with varying concentrations of TNF-α for 3–24 h; CCL5 expression was assessed by qPCR. Each data point represents the mean ± SD of three replicate cultures

**Fig. 2:**
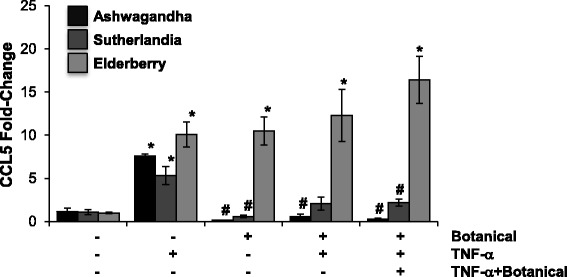
Ashwagandha prevents TNF-α-induced CCL5 gene expression. Confluent NRK-52E cells were treated for 24 h with water-soluble extracts of ashwagandha, sutherlandia or elderberry (1:100). After 24 h, cells were challenged with 10 ng/ml of TNF-α for 24 h alone or in the presence of the botanical extract. CCL5 gene expression was determined by qPCR. Each data point represents the mean ± SD of three replicate cultures, * indicates a significant difference as compared to control (no botanical, no TNF-α), # indicates a significant difference from TNF-α-challenged cultures

Initial experiments were designed to determine an optimal concentration of TNF-α to induce CCL5 expression; published studies have shown that NRK-52E cells respond to 10 ng/ml for 6–48 h [43, 44]. In our experiments, higher concentrations (100 or 500 ng/ml) failed to substantially enhance CCL5 gene expression, while results with 50 ng/ml were highly variable (Fig. 1). A concentration of 10 ng/ml for 24 h induced an approximate 10-fold increase in CCL5 gene expression that was not seen at early (3 or 6 h) timepoints (Fig. 1). We then screened water-soluble extracts of ashwagandha, sutherlandia, or elderberry at a dilution of 1:100. We used two approaches – cells were exposed to the botanical extracts for 24 h prior to 10 ng/ml TNF-α challenge alone or in the continued presence of the botanical for 24 h. Interestingly, elderberry alone induced CCL5 expression to the extent of TNF-α, while pretreatment and co-treatment with sutherlandia attenuated TNF-α-induced CCL5 expression (Fig. 2). Ashwagandha, however, completely ablated the induction of CCL5 by TNF-α when cells were pretreated or cotreated (Fig. 2). Interestingly, both ashwaganda and sutherlandia also inhibited basal CCL5 gene expression in NRK-52E cells.

**Fig. 3:**
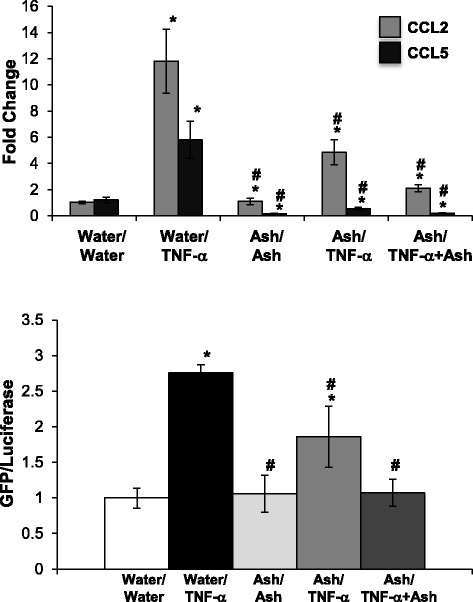
Ashwagandha attenuated TNF-α-induced CCL2 gene expression and NF-κB activation. Confluent NRK-52E cells were treated for 24 h with water-soluble extracts of ashwagandha (Ash; 1:100). After 24 h, cells were challenged with 10 ng/ml of TNF-α for 24 h alone or in the presence of the ashwaganda extract. CCL2 and 5 gene expression was determined by qPCR (*top*) while NF-κB activation was assessed using a reporter cell line (*bottom*). Each data point represents the mean ± SD of three replicate cultures, repeated twice, * indicates a significant difference as compared to control (no botanical, no TNF-α), # indicates a significant difference from TNF-α-challenged cultures

In Fig. 3, the same treatment regimens (pretreatment and pretreatment/cotreatment) were used with the ashwagandha (Ash) extracts. Pre-treatment with Ash inhibited both CCL2 and CCL5 expression induced by TNF-α. In the case of CCL2, which was induced to a higher extent than CCL5 by TNF-α, the protective effect was partial, but not complete (Fig. 3). TNF-α did not increase expression of CCL17, 19, or 21, or expression of COX-2 (data not shown). In addition, the pretreatment/co-treatment approach was most effective at attenuating CCL2 expression. Given that TNF-α induces NF-κB activation [14], and that NF-κB regulates CCL2 and 5 expression [18, 19], we used an NF-κB reporter cell line to investigate the potential of Ash to inhibit TNF-α-induced NF-κB activation. While pretreatment with Ash partially attenuated NF-kB activation following TNF-α challenge, the pretreatment/cotreatment regimen completely blocked TNF-α-induced NF-κB activation (Fig. 3). Ashwagandha did not, however, affect basal NF-kB activity parallel to the reduction in basal CCL5 expression.

**Fig. 4:**
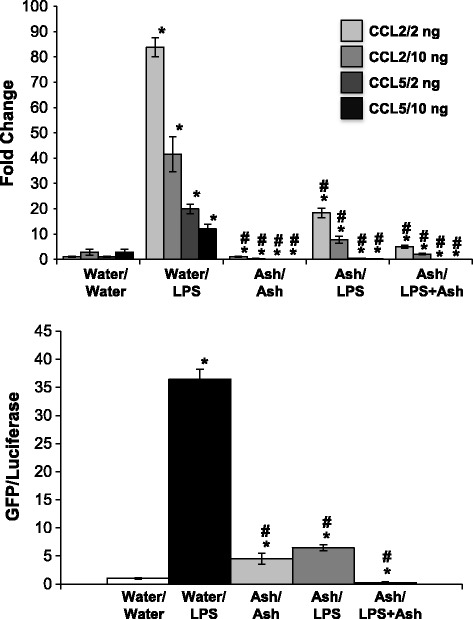
Ashwagandha attenuated LPS-induced CCL2 and CCL5 gene expression and NF-κB activation. Confluent NRK-52E cells were treated for 24 h with water-soluble extracts of ashwagandha (Ash; 1:100). After 24 h, cells were challenged with 2 or 10 ng/ml of LPS for 24 h alone or in the presence of the ashwaganda extract. CCL2 and 5 gene expression was determined by qPCR (*top*) while NF-κB activation was assessed using a reporter cell line (*bottom*). Each data point represents the mean ± SD of three replicate cultures, repeated twice, * indicates a significant difference as compared to control (no botanical, no LPS), # indicates a significant difference from LPS-challenged cultures

To ensure that the effects of ashwagandha were not limited to TNF-α, we then challenged cells with LPS using the same approach. NRK-52E cells have been challenged with 10–100 ng/ml LPS for 24 h [45]. We chose to use concentrations at the lower end of this range; 2 or 10 ng/ml LPS is associated with a robust increase in both CCL2 and CCL5 gene expression at 24 h (Fig. 4). Interestingly, the response is not dose-dependent in that the response was greater using 2 ng/ml. Pretreatment with Ash attenuated LPS-induced CCL2 and prevented CCL5 expression, while the pretreatment/cotreatment strategy further attenuated CCL2 expression and CCL5 expression was completely inhibited (Fig. 4). The pre-treatment with Ash attenuated LPS-induced NF-κB activity, while the pretreatment/cotreatment strategy prevented the effect (Fig. 4).

## Discussion

The identification of specific strategies to slow the progression of chronic renal dysfunction, or to attenuate acute injury, has been a frustratingly slow process. Given the role of inflammation in injury and progression to dysfunction via pro-fibrogenic mechanisms, strategies to attenuate inflammation are promising therapeutics. There are a number of studies suggesting that botanicals are anti-inflammatory [29–31, 36]; however, the molecular pathways targeted by many of these remains poorly defined. In our studies, we developed an in vitro model to screen botanical extracts for anti-inflammatory cells in a rat proximal tubular epithelial cell line, NRK-52E, since the proximal tubular epithelium is both a source [5] and target of inflammation in the kidney. In these studies, cells were challenged with a pro-inflammatory stimuli either after exposure to the botanical, or simultaneously with the botanical.

Interestingly, an elderberry extract, which has reported anti-inflammatory effects [46], induced CCL5 expression to a similar extent as TNF-α in our model. A sutherlandia extract partially attenuated TNF-α-induced CCL5 gene expression, but ashwaganda completely inhibited the TNF-α effect on CCL5 expression. This anti-inflammatory effect of ashwagandha is further supported by our findings that the extract attenuates TNF-α and LPS-induced CCL2 expression and NF-κB activation, and inhibited LPS-induced CCL5 expression. Ashwagandha has been reported to be renoprotective in several studies, mainly as an antioxidant. Ashwagandha is renoprotective against bromobenzene-induced nephrotoxicity [35], gentamicin-induced nephrotoxicity [34, 47] and dehydration-induced oxidative stress [33]. Mechanistically, it has a positive effect on glutathione peroxidase, superoxide dismutase, and catalase levels [34].

An interesting finding was the ashwagandha decreased basal expression of CCL5, but did not affect basal CCL2 expression or NF-kB activation. The impact of ashwaganda on CCL2 expression paralleled the effects on NF-kB activation, i.e. attenuated TNF-α- or LPS-induced increases. These data suggest that ashwagandha affects other signaling pathways which regulate CCL5 expression; candidates include the p38 MAPK pathway which has been shown to inhibit CCL5 in smooth muscle cells [48]. Withaferin A, a bioactive component of ashwagandha [49], has been shown to increase p38 activity [50], as has another component of ashwagandha, withanolide D [51].

There are a number of papers demonstrating that withaferin A inhibits NF-κB signaling [52, 53]. Withaferin A also inhibits NF-κB activation by inhibiting IKKb catalytic activity [54]. In addition, there is data demonstrating that withaferin A inhbits TNF-α-induced effects in endothelial cells [55]. We examined withaferin A in our model system, however, at concentrations (1 μM) and timepoints (2 h) previously reported [54, 55], withaferin A induced substantial cytotoxicity in our cells (data not shown). As such, withaferin A induces apoptosis in a number of cell types [56, 57] putatively via down regulation of JAK/STAT3 in cancer cells [58]. Given that withaferin A is not readily-water soluble and that it was cytotoxic, our data suggest that withaferin A is not solely responsible for anti-inflammatory effects of ashwagandha. Future studies will focus on the pathway by which ashwaganda regulates both basal- and stimulus-induced chemokine expression. In addition, the confirmation of these findings in in vivo models of progressive renal injury and dysfunction represents an important progression of botanical-based renoprotection research.

## Conclusions

A water-soluble extract of Ashwagandha has anti-inflammatory effects, specifically reducing gene expresison of CCL2 and CCL5 in response to TNFα or LPS stimulation. This may be mediated, in part, via a reduction in NF-κB activity. These data suggest that Ashwagandha may represent a botanical approach for the management of renal dysfunction.
